# Contrasting Climate Sensitivity of *Pinus cembra* Tree-Ring Traits in the Carpathians

**DOI:** 10.3389/fpls.2022.855003

**Published:** 2022-06-09

**Authors:** Marian-Ionuț Știrbu, Cătălin-Constantin Roibu, Marco Carrer, Andrei Mursa, Lucrezia Unterholzner, Angela Luisa Prendin

**Affiliations:** ^1^Forest Biometrics Laboratory, Faculty of Forestry, ‘Stefan cel Mare’ University of Suceava, Suceava, Romania; ^2^Department of Land Environment Agriculture and Forestry, University of Padova, Legnaro, Italy; ^3^Department of Biology, Aarhus University, Aarhus, Denmark

**Keywords:** dendroanatomy, functional traits, inter–intra-annual climate–structure relationships, stone pine, treeline, climatic divergence

## Abstract

High-elevation ecosystems are one of the most sensitive to climate change. The analysis of growth and xylem structure of trees from marginal populations, especially the ones growing at the treeline, could provide early-warning signs to better understand species-specific responses to future climate conditions. In this study, we combined classical dendrochronology with wood density and anatomical measurements to investigate the climate sensitivity of *Pinus cembra* L., a typical European high-elevation tree species distributed in isolated patches in the Carpathians. Samples were collected from the Retezat Mountains, South-Western Romania. We analyzed ring width (TRW), maximum density (MXD), xylem anatomical traits [cell number per ring (CNo), cell density (CD), conduit area (CA), and cell wall thickness (CWT)] time series, split into ring sectors and assessed the relationships with monthly and daily climate records over the last century (1901–2015). The analysis showed a strong dependency of TRW on CNo and MXD on CWT. Summer temperature positively correlated with MXD and CWT [monthly correlation (*r)* were 0.65 and 0.48 respectively] from the early to late wood but not TRW (*r* = 0.22). CA positively correlated with water availability (*r =* 0.37) and negatively correlated with temperature (*r =* −0.39). This study improves our general understanding of the climate–growth relationships of a European high-elevation tree species and the results could be considered for forecasting population dynamics on projected changes in climate.

## Introduction

Temperatures have increased rapidly worldwide during the last decades (IPCC, 2021) along with variation in seasonal precipitation and evaporation regimes (Konapala et al., 2020). This climate change is observed and predicted to deeply affect the structure and functioning of different forest ecosystems (Dawes et al., 2013; Büntgen et al., 2015). Among the different ecosystems, the high altitude ones, generally identified as temperature-limited environments, are experiencing warming at a faster rate than the global average (Körner, 2012; Pepin et al., 2015). Therefore, high-elevation forests are expected to be very sensitive terrestrial regions, where the effects of climate change are most likely to be observed (Harsch et al., 2009; Körner, 2012; Dawes et al., 2015). Further, the frequency, duration, and severity of the extreme climatic events are expected to increase in the future with additional consequences on the productivity, function, and distribution of forests worldwide. Recently, a trend of shifting vegetation belts toward higher altitudes and latitudes has occurred at the global scale (Körner, 1998; Harsch et al., 2009), together with an increase in productivity and photosynthetic activities (Grace et al., 2002; Carlson et al., 2017; Cazzolla Gatti et al., 2019). These changes have been mainly attributed to the effects of increasing anthropogenic greenhouse gas emissions on global temperatures (Cannone et al., 2007; Körner, 2012). Still, it is not clear what the long-term implications of such plant responses are, nor which structural adjustments will primarily allow trees to acclimate to environmental changes. For this reason, marginal populations, such as the ones growing at the elevational edge of a species’ distribution, are of particular interest. Compared to populations growing within their optimum (Fritts, 1976), marginal populations allow for investigation of climate–growth relationships and for gaining insight into inter- and intra-specific responses of trees growing at their physiological limits. The improved understanding of these plant responses could allow for forecasting the future dynamics of these populations (Hampe and Petit, 2005).

To have accurate forecasting, we need long-term data sets. Tree-ring data give a long-term, retrospective quantification of annual growth of trees, from different sites and species (Fritts, 1976; Cook and Kairiukstis, 1990), and enables identification of the factors that mainly determine tree growth (D’Arrigo and Jacoby, 1993; Mäkinen et al., 2003; Bouriaud et al., 2005). Additionally, wood density reflects a plant’s carbon accumulation in the xylem (Rathgeber, 2017), giving insight to the plant’s ecology, including functional physiology, mechanical properties, architecture, and climate responses (Hacke et al., 2001; Chave et al., 2009; Rathgeber, 2017). Tree-ring width and wood density have been the most widely used tree-ring features in dendro-climatological and -ecological studies. They are commonly used environmental proxies, that effectively record the climate signal (e.g., maximum density MXD), and they are also used to calibrate models that predict net primary production (Babst et al., 2014; Klesse et al., 2018). Still, a better understanding of plant responses to ongoing climate change requires insight into the physiological growth responses at a finer resolution (e.g., intra-annual). Therefore, coupling dendrochronology with tree-ring anatomy adds a “time component” to the functional mechanisms and plasticity of xylem formation (Fonti et al., 2010). This aids in identifying how wood anatomical adjustments determine variation in growth and density and can improve the interpretation of how they are connected and climatically controlled (Björklund et al., 2017). Thus, investigating how tree xylem structures and their associated functions change over time and in relation to environmental variability is of high importance to improve the ecophysiological understanding of the process of growth and to infer potential marginal population responses under different climate change scenarios.

Dendroanatomy is an emerging field that specifically focuses on the quantitative assessment of xylem tissues, cells, and derived metrics or traits linked to specific functional roles. This approach is based on the fact that the xylem structural adjustments are permanently recorded and chronologically archived in the structure of tree rings (Fonti et al., 2010), thus allowing retrospective analysis of the structure–function responses of trees to climate variability (Fonti and Jansen, 2012). Therefore, wood anatomical features (e.g., lumen area, related to hydraulic efficiency or cell wall thickness, related to carbon costs), localized at a certain position within yearly dated annual growth rings are linked to the time of their formation and become useful proxies to quantify long-term tree structural–functional responses and growth dynamics at an unprecedented time resolution (Fonti et al., 2010; Pritzkow et al., 2014; Baas et al., 2016; Prendin et al., 2017; Björklund et al., 2020).

The recent methodological progress in sample processing and image analysis allow for, for example, an increasing in the number of automatically measured tracheids of ~10- to 20-fold (Gärtner and Schweingruber, 2013; Von Arx et al., 2016; Prendin et al., 2017). Thanks to this, studies that combined tree-ring proxies at both annual and intra-annual resolution added depth to the inferences and improved our understanding of plant responses to climate and environmental variability (Panyushkina et al., 2003; Ziaco et al., 2014; Lange et al., 2020). Despite such progress, studies evaluating the responses of marginal populations at high elevation are scarce (Carrer et al., 2018).

A limited number of European high-elevation stands are characterized by the presence of the glacial relict stone pine (*Pinus cembra* L.; Caudullo et al., 2017). Isolated populations are growing in the Alps and Carpathians (Blada, 2008; Saulnier et al., 2011; Beloiu and Beierkuhnlein, 2019) as a consequence of climatic fluctuations, such as glacial/interglacial periods, together with species competition and anthropogenic disturbances that occurred in the last millennia (Ali et al., 2005). However, the future dynamics of these isolated populations are still uncertain as they could both expand their range due to the limitation of anthropogenic pressure and increasing temperature (Vittoz et al., 2008) or retreat, due to water limitations and competition with other taxa (Lyu et al., 2019). Despite being very suitable for investigating the climate–growth relationship, as they are rarely affected by biotic disturbances (e.g., defoliators and bark beetle outbreaks) compared to European larch (*Larix decidua* Mill.) or Norway spruce (Baltensweiler, 1993; Carrer et al., 2007; Saulnier et al., 2011), to our knowledge, only a few studies assessed the long-term intra annual climate sensitivity of this typical treeline species at the edge of its distribution (Carrer et al., 2018) and none have investigated it in combination with wood density measurements (that are rarely performed in this species).

In this study, we used a multiproxy approach to investigate the mechanism that regulates xylem growth of *Pinus cembra* L. at high elevation in the Carpathians, Romania. Specifically, we combined the classical dendroecological measurements (TRW, MXD) with dendroanatomical ones (e.g., cell density, cell number, conduit area, and cell wall thickness) to: (i) gain insight into the anatomical basis of tree-ring width and wood density; (ii) identify the key factors that determine stone pine growth and structural variability at inter- and intra-annual resolution; (iii) and shed light on the mechanism that regulates xylem growth formation of this species at the easternmost margin of its distribution area.

## Materials and Methods

### Study Site and Climate

**Figure 1:**
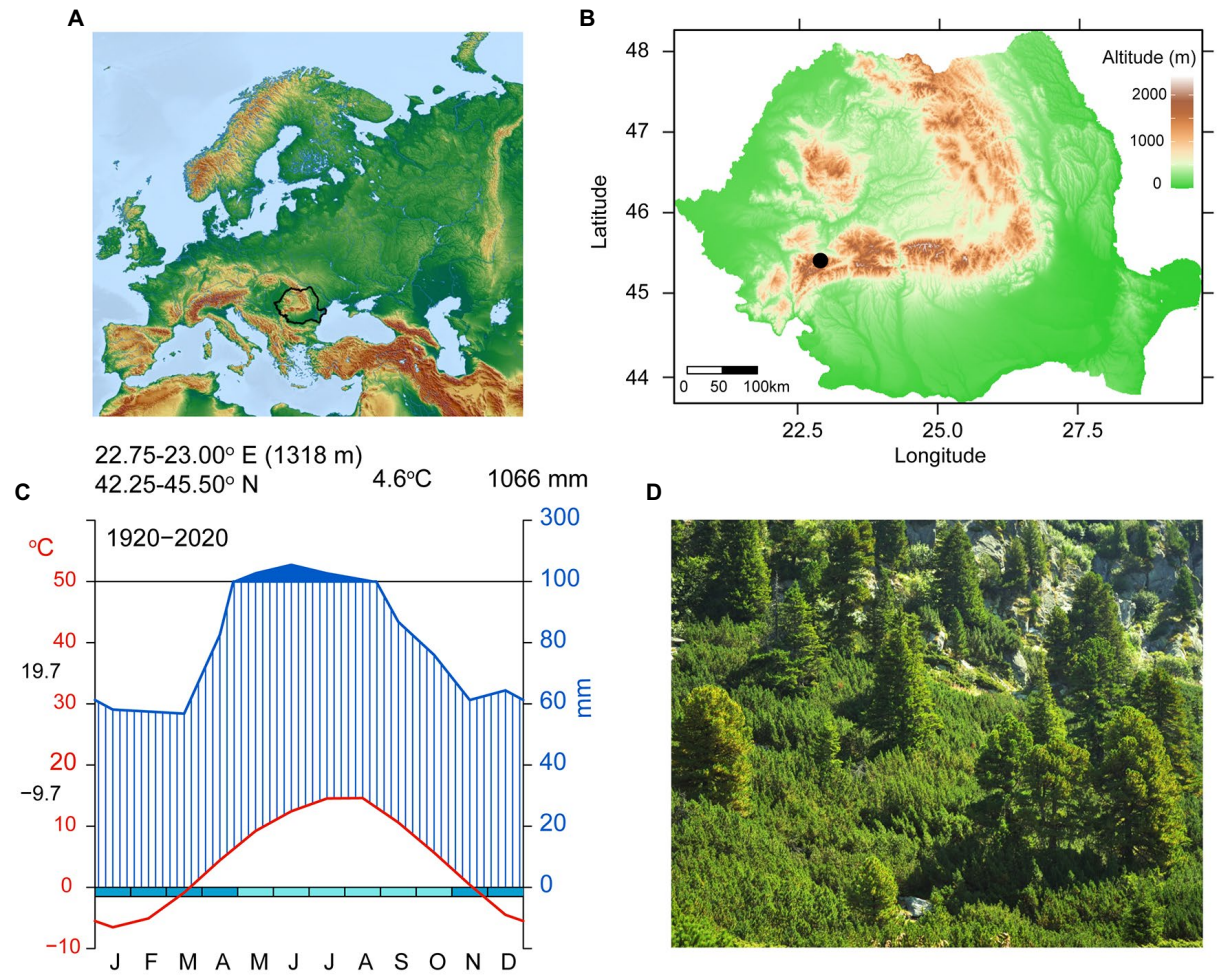
Site location **(A)** Map of Europe—black contour is Romania; **(B)** Map of Romania—black dot shows Retezat National Park location; **(C)** Walter–Lieth Climodiagram showing mean monthly precipitation (blue, mm), mean monthly temperature (red, °C), months in which frost events are likely to occur (light-blue and turquoise boxes) and wet periods (dark-blue filled areas) based on grided data; **(D)** General view of the sampling site.

The study site is represented by a natural timberline (1,700–1,800 m) of stone pine stands, located on the north-facing slope in the Retezat National Park in the South-Western Carpathians (Romania; 45^o^39′ N, 22^o^89′ E; Figure 1). The soil is shallow and the geological structure complex, mostly composed of crystalline rocks and limestone. The mean annual temperature is 4.6°C with monthly values ranging from −9.7°C in January to 19.7°C in July. Mean multiannual precipitation is 1,066 mm/year, with a peak of 156 mm/month in June (Figure 1C). Climate records used in this study were obtained from the CRU TS4.04 (Harris et al., 2020; monthly and self-calibrating Palmer Drought Severity Index (scPDSI), 1901–2015) from the closest grid points to our study region (22.50–23.00°E/45.00–45.50°N) and Rocada dataset (Dumitrescu and Birsan, 2015, daily, 1961-2013) from the closest grid points to our study region (22.80–22.90°E/45.25–45.50°N).

### Sample Collection and Processing

One increment core per tree was extracted from 28 dominant, isolated, and undamaged mature stone pine trees in June 2015. The increment cores were collected at breast height following standard procedure (Schweingruber et al., 1990) using a Pressler borer and later stored in paper straws. To perform densitometric measurements, a one-millimeter-thick longitudinal lath of wood was cut from the middle of each core, paying attention to the tracheid orientation. To obtain the MXD time series, the laths were boiled for 48 h in a Soxhlet extractor with 98% ethanol following standard protocol (Wang et al., 2002; Pritzkow et al., 2014). The radiographic images were obtained with an Itrax MultiScanner (Cox Analytical Systems, Gothenburg, Sweden) in a climate-controlled medium (50% relative humidity and 20°C) using the following settings for the X-ray tube: voltage to 30 kV, current to 50 mA, and exposure time equal to 50 ms. A 16-bit, grey level, radiographic (X-ray) digital image was obtained for each sample at 1270 dpi resolution. The maximum density (MXD) was measured using WinDENDRO density version software (Regent Instruments, 2018). Tree-ring widths (TRW) were measured to the nearest 0.001 mm on the flat surface of the remaining sides of the cores using the LINTAB system and TSAP 0.53 software (Rinn, 2003). Measurement and dating accuracy were then checked using COFECHA (Holmes, 1983). After TRW and MXD measurements, a subset of nine samples without any visible defects or cross-dating issue were selected for anatomical analysis. These nine laths left after MXD samples preparation were split into 4–5 cm long pieces, and from each one, a thin (10 μm) transversal section was cut using a rotary microtome (Leica, Heidelberg, Germany). The resulted microsections were double-stained with safranin (0.8 g in 100 ml distilled water) and astrablue (0.5 g in 100 ml distilled water +2 ml acetic acid; Gärtner and Schweingruber, 2013), and mounted on permanent slides with Eukitt (BiOptica, Milan, Italy). Anatomical images were captured using the D-sight 2.0 System (Menarini Diagnostics, Florence, Italy) at 100x magnification corresponding to a resolution of 1.99 pixels/μm and were analyzed with ROXAS v3.0.250 (von Arx and Carrer, 2014; Prendin et al., 2017). Anatomical analysis was performed on >40 radial tracheid’s rows per ring and a total of 2.5×10^6^ tracheids were measured. To increase the temporal resolution without compromising the statistics regarding sample size (i.e., cell number; Castagneri et al., 2017), based on the information of tracheid’s positions within each ring, we divided each ring into 10 ring sectors (with the 1st and 10th sectors corresponding, respectively, to the tracheid formed at the beginning and at the end of the growing season (Carrer et al., 2014). We finally obtained a time series for the whole ring and for each decile ring sector of the following anatomical parameters: cell density (CD; as cell number divided by the ring area), cell number (CNo; number of conduit per ring, standardized to a tangential width of 1.5 mm to account for size differences in the images collected, similarly to Castagneri et al. (2015), conduit area (CA), radial and tangential cell wall thickness (rCWT/tCWT), and mean cell wall thickness (CWT).

### Statistical Analysis

All TRW, MXD, and anatomical series were standardized to remove the typical age-size trend (Supplementary Figure 1; Enquist, 2002; Carrer et al., 2014; Prendin et al., 2018) using a cubic smoothing spline with a 50% frequency cutoff response of 100 years (Cook and Kairiukstis, 1990; Castagneri et al., 2015; Carrer et al., 2018). The residual autocorrelation was removed using an autoregressive model and the mean chronologies were obtained by using bi-weight robust mean (Supplementary Figure 2; Fritts, 1976; Cook and Peters, 1997). We calculated the following descriptive statistics for both raw and detrended chronologies: mean sensitivity (MS), an index of the mean relative change between trait values in consecutive years, to assess the high-frequency variations in the chronologies; mean series inter-correlation (Rbar), and the expressed population signal (EPS; Fritts, 1976; Briffa and Jones, 1990) to estimate the level of year-by-year growth variations shared by trees in the same site (Table 1).

**Table 1:** Main statistical parameters for raw and detrended xylem traits chronologies.

Trait	Number of cores	First year	Last year	MA ± SD	MV ± SD	RAW chronology			Detrended chronology	
						*rbar*	EPS	MS	*rbar*	EPS
CD	9	1735	2015	174 ± 60	1110.2 ± 102.0	0.20	0.58	0.06	0.13	0.47
CA					476.5 ± 58.4	0.19	0.57	0.10	0.20	0.58
tCWT					3.1 ± 0.2	0.23	0.63	0.05	0.32	0.72
rCWT					3.3 ± 0.1	0.24	0.64	0.05	0.31	0.72
CWT					3.2 ± 0.2	0.25	0.66	0.05	0.33	0.73
CNo					1711 ± 700	0.13	0.46	0.18	0.14	0.47
MXD		1735	2014	180 ± 55	0.9 ± 0.1	0.23	0.63	0.09	0.33	0.74
TRW					1.4 ± 0.5	0.17	0.54	0.18	0.19	0.58
MXD all	28	1,684	2015	225 ± 62	0.7 ± 0.1	0.27	0.87	0.09	0.30	0.89
TRW all		1,684	2015		1.1 ± 0.6	0.18	0.81	0.18	0.24	0.86

CD, Conduit density (number·mm^−2^); CA, Conduit area (μm); *tCWT, Tangential cell wall thickness* (μm); *rCWT, Radial cell wall thickness* (μm); *CWT, mean Cell wall thickness* (μm); CNo, Conduit number (number of conduit measured in the analyzed ring area); MXD, Maximum wood density (g·cm^−3^); TRW, Tree-ring width (mm); MXD all and TRW all—Maximum wood density and tree-ring width of all the 28 cores; cores—number of cores; MA, mean age; MV, mean values for each parameter; SD, standard deviation; rbar, inter-series correlation; EPS, expressed population signal; MS, mean sensitivity.

To investigate the association between TRW, MXD, and xylem traits chronologies, the residual chronologies were grouped employing hierarchical cluster analysis (HCA; Ludwig and Reynolds, 1988) based on Ward’s minimum variance criterion (Everitt et al., 2011). Moreover, to verify the grouping consistency at intra-annual resolution, a cluster per ring sector was performed using TRW and MXD and the anatomical parameters time series.

To identify the limiting factors of tree-ring formation, the climate/growth and structural relationships were quantified using bootstrap correlations with the R packages *treeclim* (Zang and Biondi, 2015) and *dendroTools* (Jevšenak and Levanič, 2018). In particular, the analysis tested the correlation between temperature, precipitation and scPDSI, monthly/daily climate records, and TRW, MXD, and xylem traits chronologies. Monthly correlations were computed from June of the previous year to September of the current year whereas, with the daily climate records, we kept the same time span adopted for the monthly climate–growth relationship, but we first averaged the temperature and precipitation series in a 15-day windows, then correlations were computed between June of the previous year to September of the current year shifting the time window at a daily step (Carrer et al., 2017, 2018). Three distinct 40-yr periods (1901–1940, 1941–1080, and 1981–2013) were used in order to identify possible shifts in the climate growth relationship.

## Results

### Tree-Ring Width Maximum Density and Xylem Traits Chronologies

The mean series length is 225 ± 62 years, with a mean ring width of 1.10 ± 0.6 mm· and a MXD of 0.7 ± 0.1 g·cm^−3^. The subset of the nine samples included relatively younger individuals (174 ± 60 years), wider annual rings (1.40 ± 0.5 mm), and higher MXD (see Table 1; Supplementary Figure 2). The averaged cell density was 1110.2 ± 102.0 cells·mm^−2^, while the mean conduit area (CA) corresponded to 476.5 ± 58.4 μm^2^·, and the anatomical parameters related to cell wall thickness, presented similar values, ranging from 3.3 ± 0.1 (rCWT) to 3.1 ± 0.2 μm (tCWT) with a mean of 3.2 ± 0.2 μm (CWT). The strength of the common signal and the quality of the chronologies assessed using Rbar and EPS statistics found that MXD and cell wall thickness traits showed generally higher values compared to TRW, CD, and CA both in the RAW and detrended chronologies (Table 1). The mean sensitivity for TRW and CNo was higher (0.18) compared to MXD, CD, CA, and cell wall thickness traits (0.05; Table 1). Similar results were obtained when assessing the descriptive statistics of xylem traits chronologies at intra-annual resolution (10 ring sectors; Supplementary Table 1). Nevertheless, the signal strength between chronologies increased from first to last ring sectors for all anatomical traits (except for cell density).

### Relationships Between Tree-Ring, Maximum Density, and Anatomical Traits

**Figure 2:**
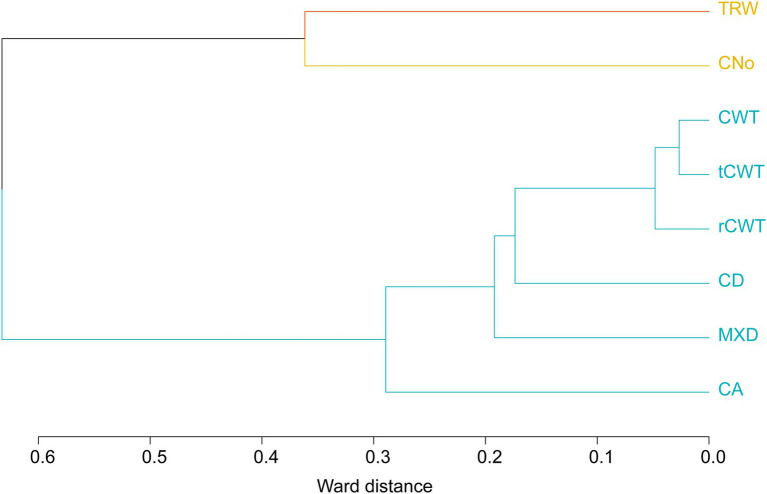
Hierarchical cluster analysis using TRW, MXD and anatomical parameters (with yellow—first cluster, blue—second cluster).

The hierarchical cluster analysis showed a strong relationship between CA, MXD, CD, and CWT. TRW and CNo were more independent in comparison with other variables (Figure 2 and Table 1). Cell wall thickness chronologies (rCWT, tCWT, and CWT) formed two relatively closed (<0.1) clusters referring to the Ward distance followed by CD, MXD (<0.02), and finally CA (<0.3), while TRW and CNo formed the furthest away (>0.3) cluster on the axis (Figure 2). The HCA analysis based on intra-annual resolution chronologies (10 ring sectors) showed similar results compared to the HCA based on chronologies at annual resolution (see Supplementary Figure 3). Due to the comparable statistical parameters and similar associations between the rCWT, tCWT, and CWT chronologies, further analysis focus on the CWT chronologies only.

### Relationships With Climate

**Figure 3:**
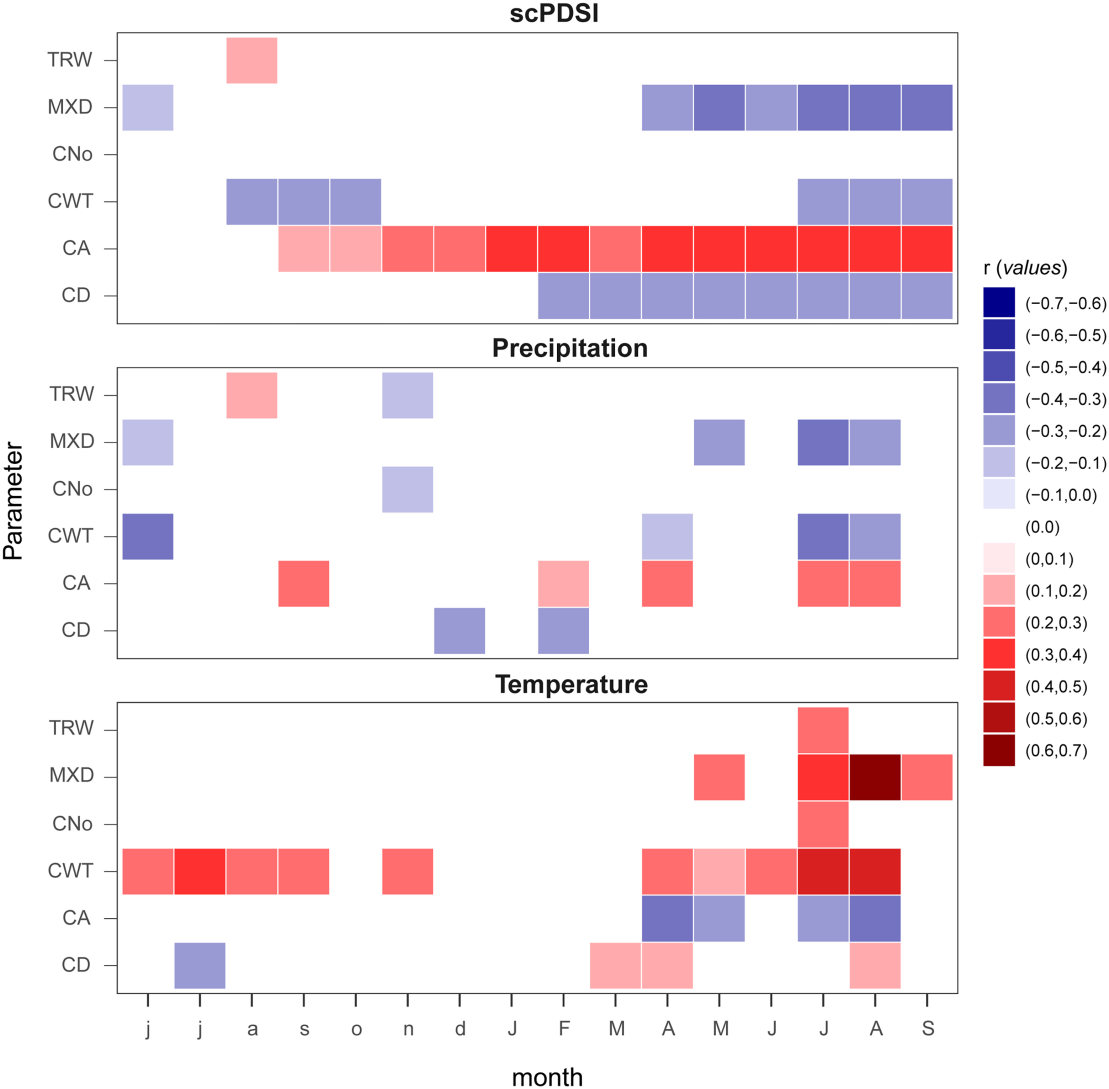
Climate growth relationships computed between monthly climatic parameters (mean temperature, accumulation of precipitation, and scPDSI; from 1901 to 2015) and TRW, MXD, and anatomical parameters (CA, CNo, CD, and CWT). Lower case letters show the previous year and capitalized the current year.

Correlations computed with monthly climate records highlight TRW and CNo as the least sensitive parameters with very similar cluster associations, while, the temperature is the main factor, positively influencing xylem density (MXD and CWT). High precipitation amount (June previous and current year summer) and high self-calibrating Palmer Drought Severity Index (scPDSI) values seemed to negatively affect MXD and CWT (Figure 3).

**Figure 4:**
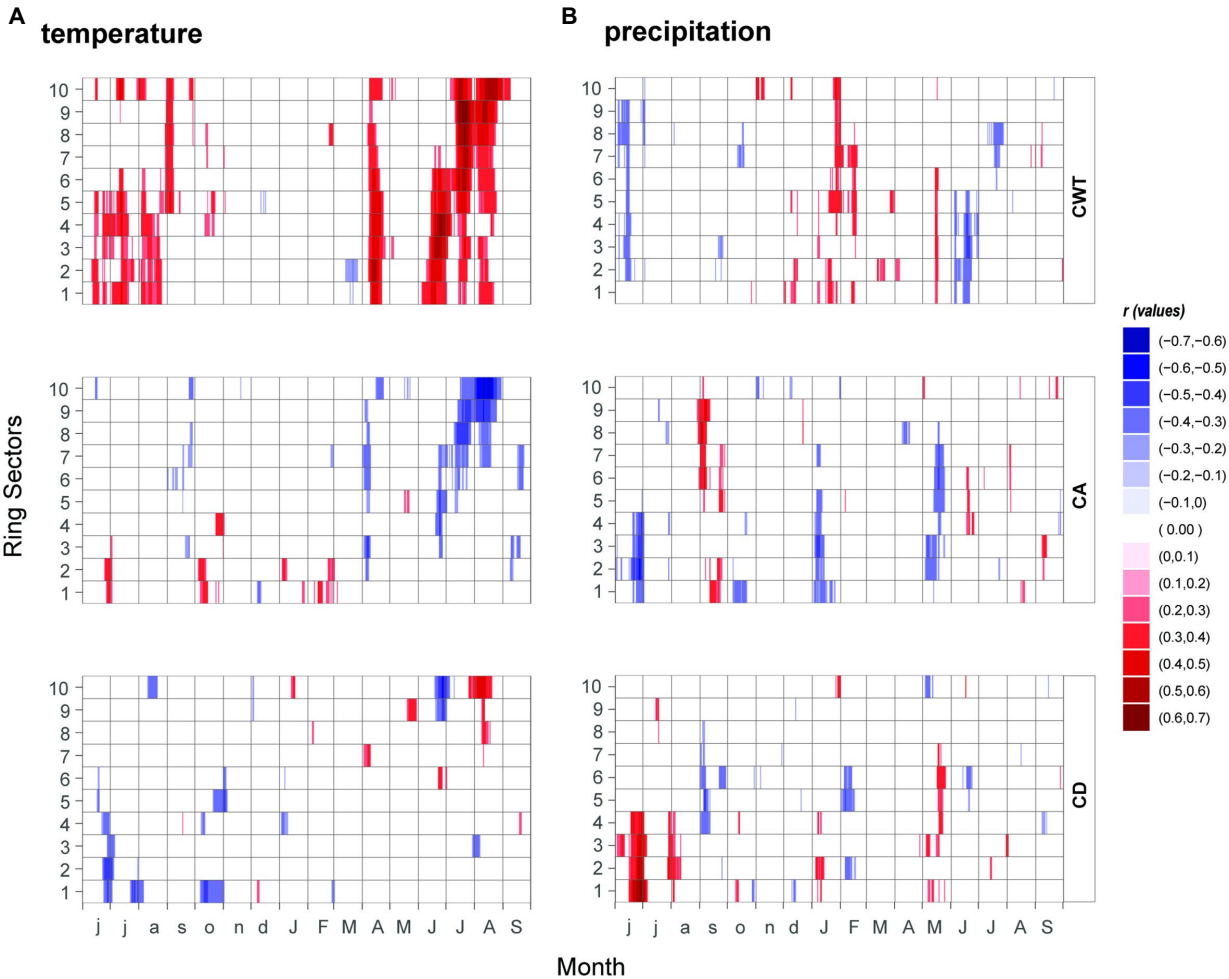
Relationships between **(A)** temperature, **(B)** precipitation and xylem anatomical traits (CA, CD and CWT) from 1961 to 2013. Correlation coefficients were assessed at 15-day windows, represented by sliding daily steps from previous June to current September and coded according to the color key on the right.

The higher resolution of the 15-day running correlations showed more detailed information (Figures 4A,B). CD showed the least significant correlations with temperature, randomly scattered, mostly during the summer to autumn months. CA showed a negative correlation with temperature starting from the third ring sector from mid-June to the end of August in the last ring sectors. CA is also negatively correlated with the temperatures of April. CWT showed an overall stronger correlation value with respect to CA, with a positively significant response at the beginning of the growing season (April–May period) in all ring sectors. During the growing season the correlation shifts from sectors 1 to 6 in the early June–July period, to sectors 4 to 10 in late June–late August, with a peak for the 10th ring sector in August (*r* = 0.63). Additionally, when splitting the last century into three equal periods (see Supplementary Figure 4), a clear weakening of the climatic signal emerged in all tree-ring parameters. In addition, the climate signal overturned in almost all the climate–growth and structural relationships, shifting from being positive in the first period to negative in the last one. MXD was one exception, with consistent positive and negative relationships throughout all the periods, despite experiencing a weakening of its climatic signal (especially for temperature) through time. Another exception was CA showing strong correlation with the increase of drought limitation in the most recent periods, while in the early period there was a non-significant relationship between CA and scPDSI.

Similarly, to the monthly analysis, the correlations between precipitation and xylem traits chronologies resulted in scattered points and were significant for a relatively short time window, indicating that precipitation plays a minor role here. Still, late spring precipitation showed the stronger signal in the first half of the ring sectors for CD (positive) and CA (negative). CWT shows a positive response to precipitation with higher correlations in the first six ring sectors for a very limited time window in mid-May. Negative correlations of CWT to daily precipitation are also found in June (from the 1st to the 5th sector) and July (from the 6th to the 8th sector).

## Discussion

Trees continuously increase in size during ontogeny by accumulating xylem biomass and adjusting their xylem structure to achieve an optimal balance between competing needs mostly related to water transport and mechanical support (Sauter, 1986). These xylem adjustments must also take into account all the external environmental factors and their variability with climate likely being the major one, always and pervasively present. In the ongoing climate change context, it is fundamental to deal with processes often operating at decadal and longer time scales, especially when studying long-living organisms, such as trees. The retrospective analysis of tree rings provides useful insight into long-term wood formation dynamics and related relationships with climate (Fritts, 1976), and into the role of trees and forests in the terrestrial carbon cycle (Babst et al., 2013). Within this framework, the analysis of xylem anatomical parameters, in parallel with tree-ring width and wood density, might allow insight to not just yearly and longer time scales but also at an intra-annual resolution (Fonti et al., 2010; Seo et al., 2011; Pritzkow et al., 2014). This study, by combining classical dendrochronology (ring width and maximum density) with quantitative wood anatomy (anatomical parameters related to water transport and cell enlargement (CA), mechanical support and wall thickening (CWT) and CD) allows us to better understand tree-ring structure of stone pine’s and assess the sensitivity to climate variability, shedding light on the mechanism that regulates xylem growth formation.

The HCA analysis indicated CNo as the main determinant for the TRW (Vaganov et al., 2006), confirming that wider tree rings were mostly formed by a higher number of cells, rather than larger cells (Vaganov et al., 2006; Björklund et al., 2017; Carrer et al., 2017, 2018). The CWT and MXD resulted in highly similar associations at both annual and intra-annual resolution (Figure 2 and Supplementary Figure 3), showing the strong dependency of cell wall material in determining the MXD, especially in the last ring sectors (Wang et al., 2002; Cuny et al., 2014; Pritzkow et al., 2014; Björklund et al., 2017, 2020). Furthermore, MXD and CWT chronologies showed a strong common signal compared to tree-ring width, CNo, CA, and CD. This confirms its high potential as high-resolution paleoclimate proxy (Büntgen et al., 2005; Björklund et al., 2019, 2020; Nagavciuc et al., 2019). The descriptive statistics of TRW and xylem traits chronologies from stone pine are consistent with previous studies (Briffa et al., 2001; Vaganov et al., 2006; Carrer et al., 2018).

Despite being at high elevation, where summer temperature is expected to be the most important limiting factor (Büntgen et al., 2006; Popa and Kern, 2009; Popa and Bouriaud, 2013; Nagavciuc et al., 2019; Ionita et al., 2020), in this site TRW and CNo seemed minimally influenced by temperature. The lack of climatic signal could be explained at different levels: (i) cambial activity and the maximum growth rate are more influenced by the photoperiod and are unconstrained by concurrent climate conditions (Rossi et al., 2006); (ii) this unexpected result could be a consequence of the Atlantic and Mediterranean climatic influences on the study site on the site (ANM, 2008) that could buffer the role of temperature as a growth-limiting factor; or (iii) a weakening of the climatic signal in the TRW has also been observed in recent decades (D’Arrigo et al., 2008; Carrer et al., 2017; Camarero et al., 2021). In this study, the negative correlations between CA and temperature could be related to the cell wall thickening process. Higher temperatures can induce the formation of cells with narrower lumen thanks to the positive stimulus on cell wall thickening but with the side effect of reducing lumen area (CA negatively associated with temperature) from earlywood to latewood, causing a denser wood and higher MXD (Cuny et al., 2014). Indeed, cell dimension could be considered the main factor of wall thickness and density changes along a ring, as cell wall thickening proceeds inside the cell whose dimension have already been fixed before the starting of the thickening process (Cuny et al., 2014; Björklund et al., 2017; Carrer et al., 2017). Temperature is generally positively associated with CWT and wood density with the resistance to cavitation (Rosner, 2013; e.g., a freeze–thaw induced embolism that can occur at high elevation). Similarly, it seems that hydraulic safety is prioritized over efficiency under droughts as CA was positively associated with scPDSI. This suggests potential xylem modification under drought scenarios (Campelo et al., 2010) since the shallow soils often associated with such treeline stands have low water holding capacity (Anfodillo et al., 1998; Oberhuber, 2004; Mayr, 2007). Therefore, we can infer that the improved growing condition, mostly related to warming in the last decades, is playing a key role in influencing the xylogenesis process and cell morphology (Schweingruber, 1996; Körner, 1998; Gruber et al., 2009).

The uphill treeline shift observed in many sites worldwide could be related to this relaxation of the limiting conditions at high elevation (Körner, 1998, 2012; Harsch et al., 2009). This significant modifications recorded at high-elevation over time implies that older trees are now growing in less constrained conditions and brings potential implication for the climatic signal encoded in the tree-ring parameters (Drew et al., 2013; see Supplementary Figure 4). Previous studies found similar contrasting results between tree-ring parameters. For example, temperature and TRW were not related, especially where trees are growing in their optimum (Poussart and Schrag, 2005; Gagen et al., 2006; Drew et al., 2013), while a strong correlation with summer temperature of MXD and CWT have been observed in similar areas (Büntgen et al., 2010; Diego Galván et al., 2015; Björklund et al., 2017). On the contrary, the negative relationship between MXD and CWT chronologies with precipitation could reflect the inverse relationship between precipitation and temperature, especially in mountain regions (Rebetez, 1996).

In this study, CWT was highly influenced by previous-year summer temperature (Frank and Esper, 2005; Babst et al., 2013), whereas CA mainly reflected the previous autumn–winter water availability conditions (Castagneri et al., 2015). Thus, the xylem structure encoded information also related to previous-year climate condition. These outcomes stressed the key role, not just of the current environmental conditions, but also of the lag effects which are usually overlooked in many investigations (Carrer et al., 2010; Babst et al., 2012). The higher detailed analysis at intra-monthly resolution computed with the daily climatic data shows significant positive correlations between temperature and CWT and a negative correlation with CA within almost all ring sectors between April and May. This probably reflects the effect of late spring temperatures on the timing of cambial activity onset and growing season duration (Carrer et al., 2017), and possibly the availability of resources stored before the wood formation (Björklund et al., 2017). Also, higher spring temperatures trigger the release of growth hormones (i.e., auxin and gibberellin; Aloni, 2015), implying an earlier onset of cambial activity (Olano et al., 2013; Carrer et al., 2017; Castagneri et al., 2017) which translates into more intense carbon assimilation (Wolf et al., 2012; Castagneri et al., 2017; Nagavciuc et al., 2020) and finally in thicker cell walls.

Different tracheids, based on their position within the ring can distinctly record different climatic time windows as shown through the increasing significant correlations between CWT and temperature from the beginning of June in the first ring sector until the start of September in the last one (Castagneri et al., 2017; Carrer et al., 2018). CWT and MXD correlations with temperature are very similar, especially when considering the last CWT ring sectors. Therefore, we suggest using CWT in dendroclimatological investigations as it represents a parameter less influenced by laboratory-dependent measuring techniques (Wang et al., 2002; Björklund et al., 2019).

## Conclusion

The combination of classical dendrochronology with quantitative wood anatomy (dendroanatomy), is a powerful tool that provides high-resolution information on structure–function tree responses over the last century. The analysis of intra-annual variation of anatomical traits, together with daily temperature and precipitation records allowed us to assemble xylem traits chronologies that maximize climatic responses compared to the classical approach, which considers just monthly climatic values and yearly resolution tree-ring data. Hence, this study contributes to the general understanding of the climate–growth and xylem structure relationship of this species on different time scales. With this, we highlighted the strong role of temperature in influencing the “carbon-sink” capacity of stone pine and gained insights into such high-elevation marginal populations under future climate scenarios.

## Data Availability Statement

Data associated with this article are deposited in the Dryad Digital Repository https://doi.org/10.5061/dryad.tqjq2bw28.

## Author Contributions

C-CR and MC initiated the collaboration project. M-IS, C-CR, and AM defined the sampling design. C-CR and AM collected samples and performed fieldwork activities. C-CR, MC, and AP designed the work. AM was responsible with the MXD sample preparation and data acquisition. M-IS carried out the dendrochronological investigations in terms of data acquisition and analyses with the help of C-CR, MC, and AP. AP was responsible for the ring sector analysis. M-IS interpreted the data results with the help of C-CR, MC, LU, and AP. C-CR processed the data and prepared all the figures with inputs from all co-authors. M-IS, MC, and AP drafted the first version of the manuscript with the help of C-CR and LU. M-IS and AP wrote the paper with input from all co-authors. The final version of the manuscript was read and approved by all the co-authors.

## Funding

C-CR was partially funded by Ministry of Research, Innovation and Digitalization within Program 1 - Development of national research and development system, Subprogram 1.2 - Institutional Performance - RDI excellence funding projects, under contract no. 10PFE/2021; C-CR and AM were partially supported by EEA Financial Mechanism 2009–2014 under the project CLIMFOR contract no. 18SEE/2014; C-CR, AM, and M-IS were partially supported by the EU cross-border project “Promote deadwood for resilient forests in the Romanian-Ukrainian cross border region” (RESFOR), no. 2SOFT/1.2/13; M-IS was partially supported by an ERASMUS+ internship in 01.03–01.06.2019 period. AP was supported by the 2017 BIRD Project of TeSAF Department University of Padova, and by Marie Sklodowska-Curie Individual Fellowship (IF) under contract number 895233.

## Conflict of Interest

The authors declare that the research was conducted in the absence of any commercial or financial relationships that could be construed as a potential conflict of interest.

## Publisher’s Note

All claims expressed in this article are solely those of the authors and do not necessarily represent those of their affiliated organizations, or those of the publisher, the editors and the reviewers. Any product that may be evaluated in this article, or claim that may be made by its manufacturer, is not guaranteed or endorsed by the publisher.
