# Supplementary material for: Contrasting Climate Sensitivity of Pinus cembra Tree-Ring Traits in the Carpathians
Source: Front Plant Sci. 2022 Jun 9;13:855003. doi: 10.3389/fpls.2022.855003 (PMC9228034; doi:10.3389/fpls.2022.855003)
Supplement: Supplementary file 1 [file Presentation_1.pdf]

Frontiers in Plant Science

Supplementary Material

Contrasting climate sensitivity of *Pinus cembra* tree-ring traits in the Carpathians

Marian-Ionuț Știrbu[1], Cătălin-Constantin Roibu[1*], Marco Carrer[2], Andrei Mursa[1], Lucrezia Unterholzner[2], Angela Luisa Prendin[2,3*]

[1] Forest Biometrics Laboratory – Faculty of Forestry – "Stefan cel Mare" University of Suceava, Universității street no. 13, 7200229, Suceava, Romania

[2] Department of Land Environment Agriculture and Forestry, University of Padova, 35020 Legnaro, Italy

[3] Department of Biology, Aarhus University, Ny Munkegade 116, 8000 Aarhus C, Denmark

*** Correspondence:**
Cătălin-Constantin Roibu
catalinroibu@usm.ro

Angela Luisa Prendin
angelaluisa.prendin@bio.au.dk

Supplementary Figure 1: Age variability of tree-ring and xylem traits as a function of cambial age, from the pith toward the bark. The panels show the age-related trends of a) Conduit area (CA), b) mean Cell wall thickness (CWT), c) Conduit density (CD), d) Conduit number, e) Tree-ring width and f) Maximum wood density (MXD). Each data point represents the trait value of a tree ring of a subset of 4 Stone pine trees with the possibility of estimating cambial age. Solid lines show the best fitting curves.

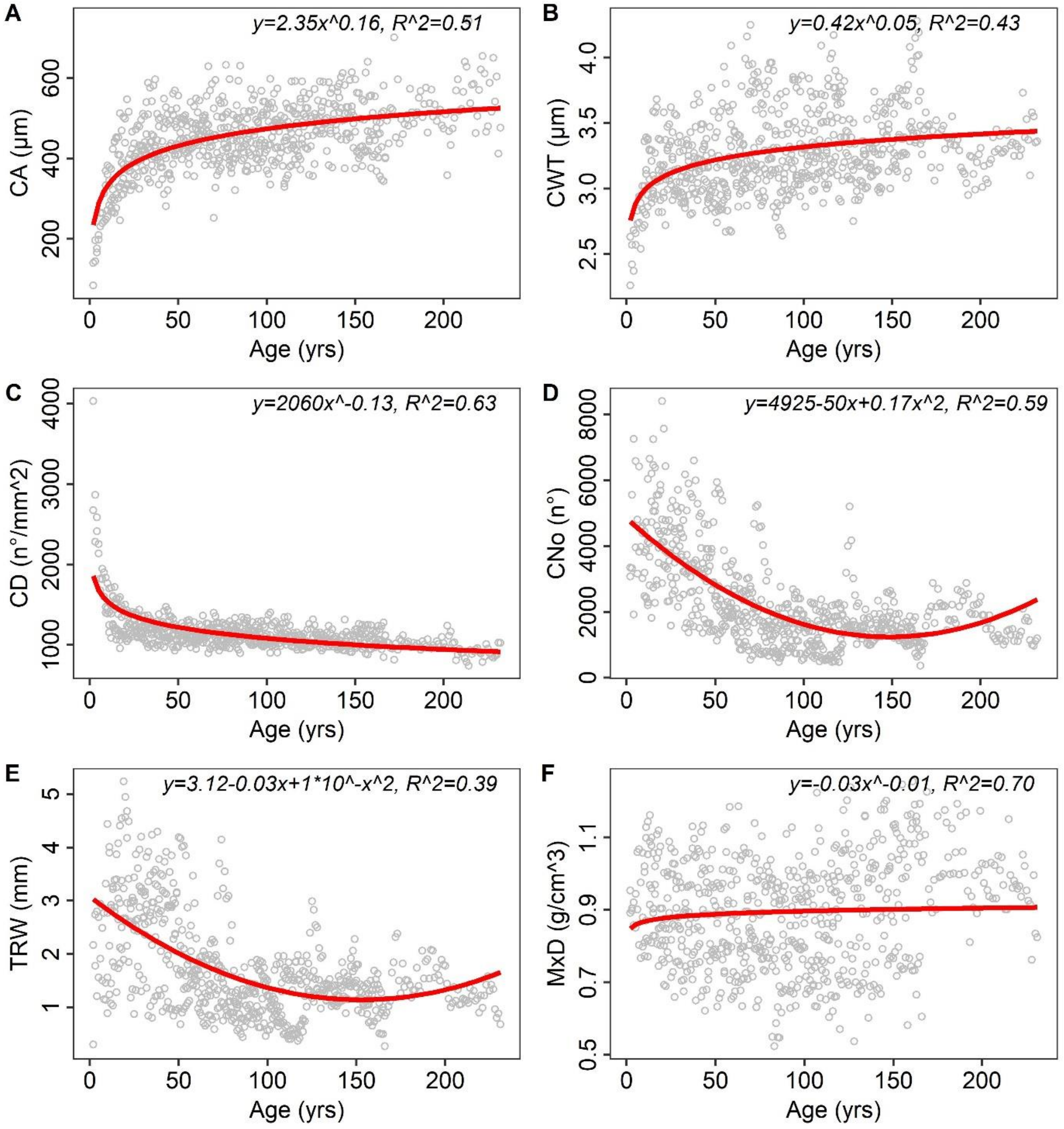

Supplementary Figure 2 Time series of the different tree-ring parameters of the nine stone pine trees analyzed in this study. Data are means (black line). The red line represents the 31-year low pass filter. The left and the right panels show raw and detrended (using a cubic smoothing spline with a 50% frequency cut-off response of 100 years) data respectively.

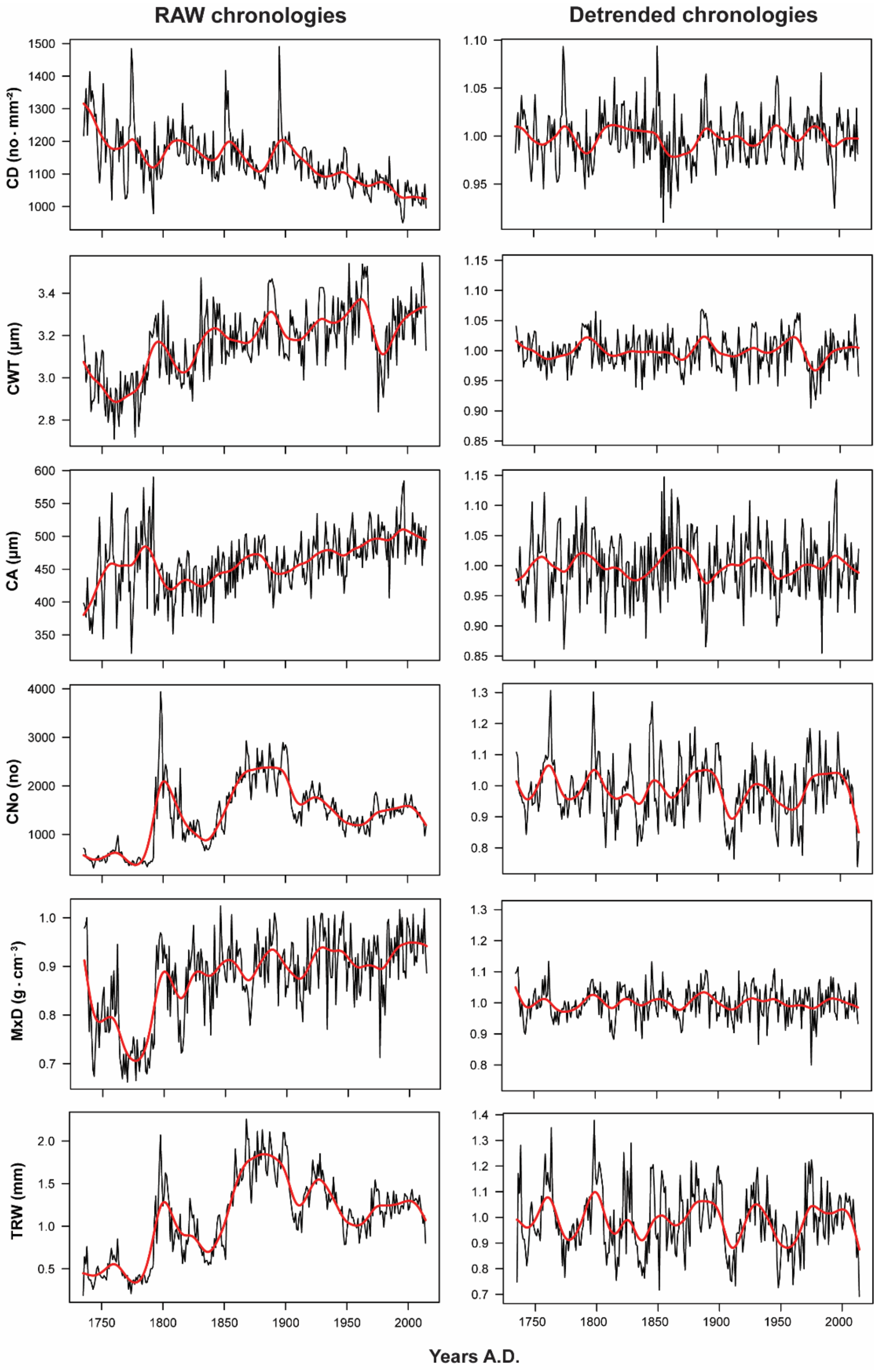

Supplementary Figure 3 Hierarchical cluster analysis using TRW, MXD and xylem traits for each sector.

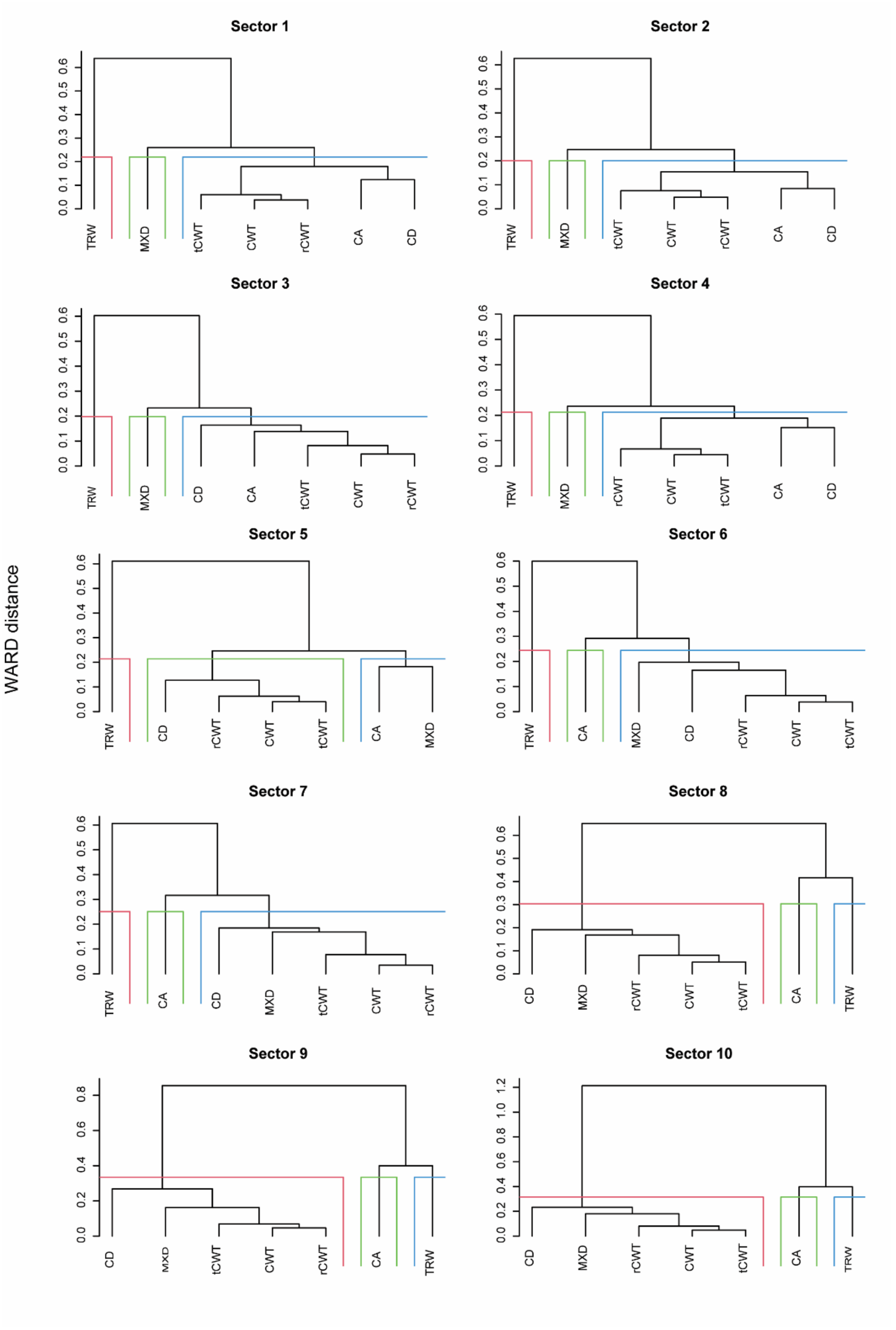

Supplementary Figure 4 Climate growth relationships computed between monthly climatic parameters (temperature, precipitation, and scPDSI) (expressed in three intervals; 1901-1940, 1941-1980, and 1981-2013) and TRW, MXD, CNo, CWT, CA and CD.

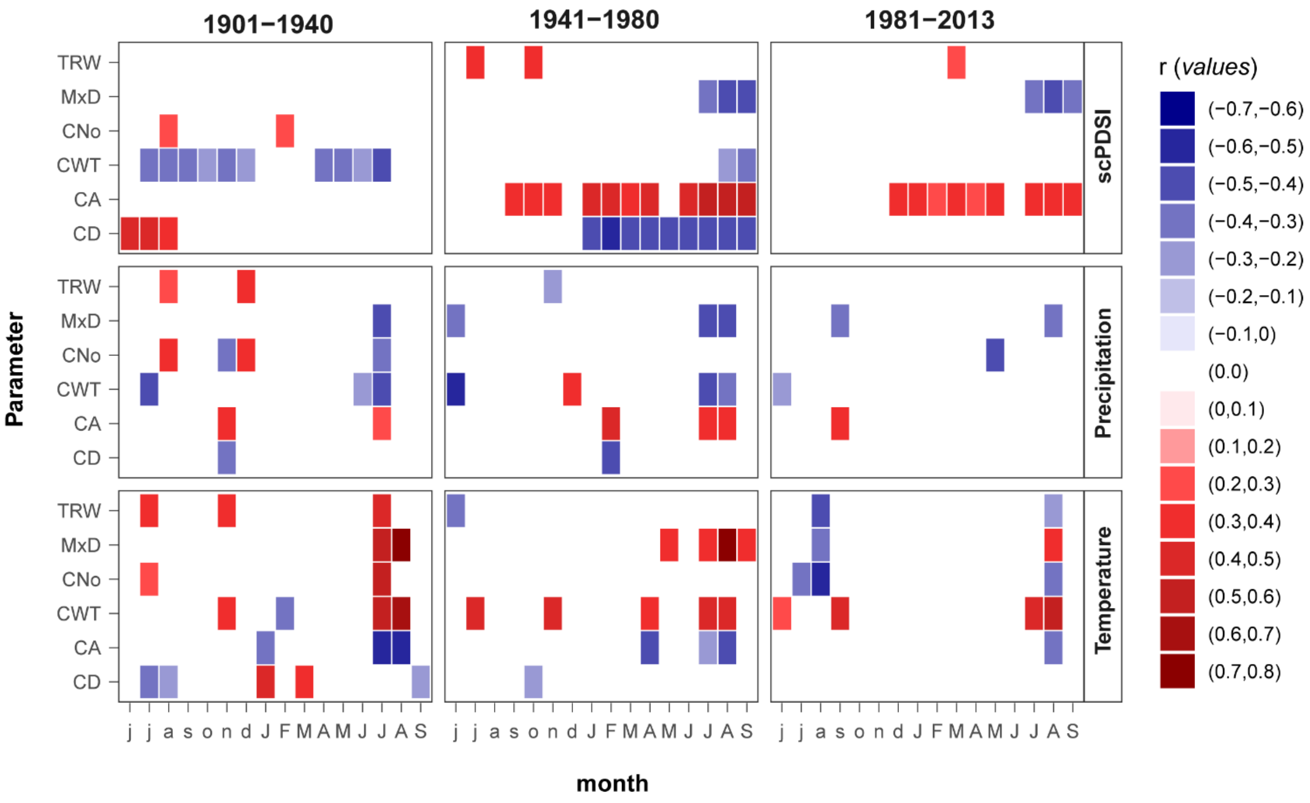

Supplementary Table 1 Main statistical parameters for intra-annual xylem trait chronologies (MV – mean values for each parameter; SD – standard deviation; rbar – inter-series correlation; EPS – expressed population signal; MS - mean sensitivity).

Anatomical trait	Sector	Statistical parameters				
		**MV**	**SD**	**rbar**	**EPS**	**MS**
rCWT	1	2.87	0.14	0.16	0.52	0.05
	2	2.89	0.14	0.19	0.57	0.05
	3	2.90	0.13	0.19	0.57	0.05
	4	2.93	0.12	0.18	0.55	0.05
	5	2.97	0.11	0.20	0.58	0.06
	6	3.04	0.12	0.21	0.60	0.06
	7	3.13	0.14	0.28	0.68	0.07
	8	3.28	0.17	0.31	0.72	0.08
	9	3.55	0.20	0.29	0.70	0.10
	10	4.08	0.26	0.27	0.68	0.10
tCWT	1	2.75	0.25	0.19	0.57	0.06
	2	2.81	0.26	0.24	0.64	0.06
	3	2.85	0.25	0.26	0.66	0.06
	4	2.91	0.25	0.26	0.66	0.06
	5	2.97	0.25	0.25	0.65	0.06
	6	3.04	0.26	0.22	0.61	0.07
	7	3.12	0.27	0.25	0.66	0.07
	8	3.23	0.27	0.29	0.69	0.08
	9	3.40	0.27	0.29	0.70	0.09
	10	3.38	0.25	0.24	0.64	0.09
CWT	1	2.83	0.19	0.20	0.59	0.05
	2	2.87	0.19	0.26	0.66	0.05
	3	2.89	0.18	0.25	0.66	0.05
	4	2.93	0.18	0.25	0.65	0.05
	5	2.99	0.18	0.24	0.64	0.06
	6	3.06	0.19	0.24	0.64	0.06
	7	3.15	0.20	0.28	0.69	0.07
	8	3.28	0.22	0.31	0.72	0.08
	9	3.49	0.22	0.31	0.71	0.09
	10	3.74	0.23	0.28	0.68	0.09
CA	1	597.18	87.33	0.07	0.29	0.12
	2	662.12	92.01	0.07	0.29	0.11
	3	663.67	90.95	0.08	0.33	0.10

	4	648.11	87.03	0.07	0.31	0.11
	5	621.03	85.81	0.10	0.39	0.11
	6	582.71	86.37	0.14	0.47	0.12
	7	534.02	84.02	0.17	0.53	0.13
	8	466.26	74.32	0.22	0.62	0.17
	9	362.54	34.47	0.24	0.64	0.21
	10	155.67	20.38	0.30	0.71	0.27
CD	1	150.31	61.62	0.12	0.44	0.19
	2	141.17	57.67	0.12	0.42	0.20
	3	142.43	59.24	0.13	0.46	0.19
	4	145.41	61.27	0.13	0.46	0.20
	5	150.33	63.73	0.13	0.46	0.19
	6	156.92	67.07	0.15	0.50	0.19
	7	165.64	71.06	0.17	0.54	0.20
	8	176.98	76.97	0.17	0.53	0.20
	9	196.28	79.96	0.14	0.48	0.20
	10	280.68	104.28	0.12	0.42	0.17
